# Modification of Oligo- and Polylactides With Macrocyclic Fragments: Synthesis and Properties

**DOI:** 10.3389/fchem.2019.00554

**Published:** 2019-08-02

**Authors:** Olga A. Mostovaya, Vladimir V. Gorbachuk, Pavel L. Padnya, Alena A. Vavilova, Gennady A. Evtugyn, Ivan I. Stoikov

**Affiliations:** ^1^Department of Organic Chemistry, A. M. Butlerov' Chemistry Institute, Kazan Federal University, Kazan, Russia; ^2^Department of Analytical Chemistry, A. M. Butlerov' Chemistry Institute, Kazan Federal University, Kazan, Russia

**Keywords:** oligolactide, polylactide, synthesis, calixarene, cyclodextrin, tetrapyrrole, porphyrin, macrocycle

## Abstract

Products of lactic acid polycondensation (poly- and oligolactic acids) are widely used as packaging materials, drug delivery agents, implants etc. Variety of their applications is caused by a number of practically important properties, e.g., biocompatibility and biodegradability, non-toxicity, and mechanical durability. Modification of these polymers with different additives allows improving their properties and extending future applications. In this manner, stability toward degradation, recognition of some substrates, extended thermal stability etc. can be improved. Macrocyclic compounds are promising candidates as modifiers. They are able to provide polymer materials with additional binding sites, impart certain orientation to spatial arrangement of polymer chains, change hydrophilic-lipophilic balance, and redox properties. The latter one can be used for assembling various electrochemical sensors and biosensors that combine steric discrimination of the analytes caused by oligolactides and highly sensitive response to their quantities caused by redox labels introduced. Different composite materials based on oligolactides as matrices for such redox labels were described in the assemblies of biosensors for drugs, pesticides, and antioxidants detection. In this mini-review, methods for the synthesis of the lactic acid oligomers and those modified with the macrocyclic fragments (porphyrin, cyclodextrin, and cyclophane) have been described. The effects of modifiers on complexation, thermal, and aggregation properties of materials are described. Analytical performance of oligolactide based sensors and biosensors has been considered with particular emphasis to the mechanism of signal generation.

## Introduction

Recently, polylactic acids (PLA) and their modification products have found increasing attention as functional materials due to non-toxicity, biodegradability, biocompatibility, and mechanical durability (Garlotta, 2001). Hydrophilicity, chemical, and thermal stability of such materials was varied by introduction of appropriate modifiers (Marcincinova-Benabdillah et al., 2001; Kumar et al., 2017). As a result, PLA was utilized in drug delivery systems [1986, Decapeptyl® (Jain et al., 2016)] and as a component of drug formulations. A particular PLA advantage is that drug can release from such a matrix for several months (Andreopoulos et al., 2000). Inflammatory reactions mentioned as a negative effect of PLA application related to the removal of the polymer degradation products can be suppressed by addition magnesium hydroxide or calcium carbonate able to neutralize lactic acid (LA) (Kum et al., 2013; Murariu and Dubois, 2016).

**Figure 1:**
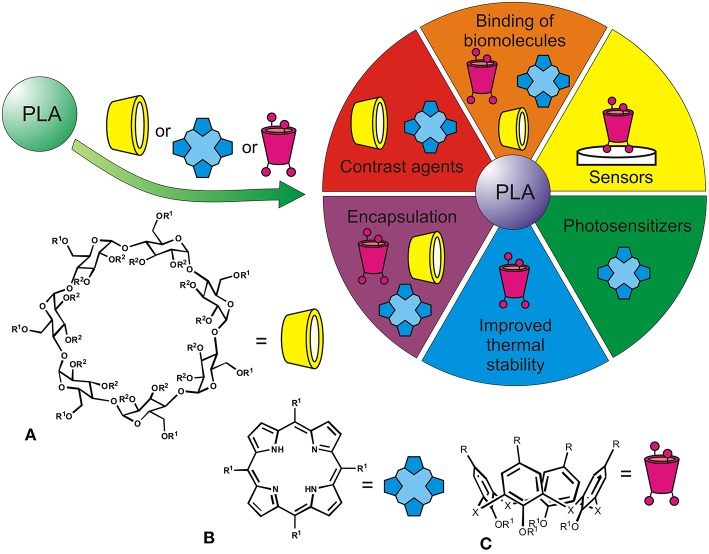
Applications of PLA functionalized with macrocyclic structures [β-cyclodextrins **(A)**, tetrapyrroles **(B)**, calixarenes **(C)**, R^1^, R^2^ indicate possible modification by PLA fragments].

The PLA is usually synthesized in three ways, LA condensation/coupling, azeotropic dehydrative condensation, and by ring opening polymerization (ROP) of lactide (Garlotta, 2001; Pretula et al., 2016; Ren et al., 2016). Polymer modification is achieved by introduction of additives containing carboxyl or hydroxyl groups and acid anhydrides into the reaction media. Recently, macrocyclic fragments have been actively studied for this purpose. They offer fixed spatial separation of the binding groups to get variety of ligands toward different molecules to be recognized (Poulsen et al., 2015; Imran et al., 2018) (Figure 1).

## Hybrids With Cyclodextrins

Cyclodextrins applied in the pharmacy, food and cosmetic industries, biotechnology are produced by enzymatic degradation of starch (van de Manakker et al., 2009; Crini, 2014) (Figure 1A). They contain spatially stable hydrophobic cavity that captures small molecules.

**Figure 2:**
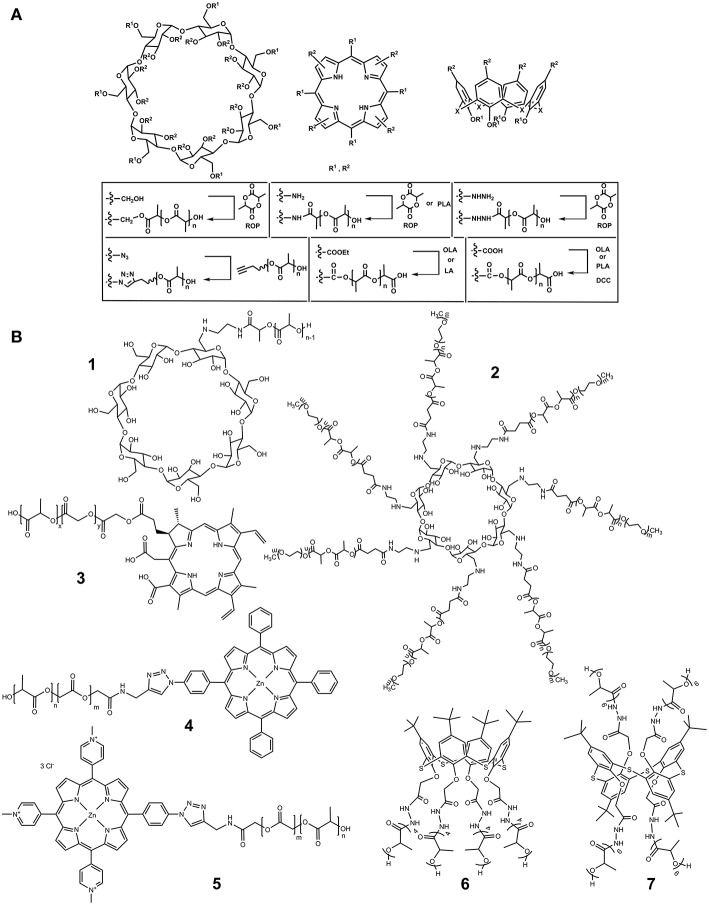
**(A)** Synthetic approaches to PLA modification by macrocycles. **(B)** Macrocycles functionalized by PLA fragments.

In 2008, one-handed lactide derivative of β-cyclodextrin (CD) was obtained by ROP of 3,6-dimethyl-1,4-dioxane-2,5-dione (lactide) in the absence of any catalyst (Shen et al., 2008; Figure 2A). Its functionalization was carried out via primary hydroxyl group farthest from the macrocycle (Figure 1A, R^1^). Introduction of oligomeric lactic acid (OLA) into the CD platform increased solubility of the product in the water. The resulting hybrid formed an inclusion complex with amoxicillin, a common antibiotic.

In the same year, copolymers with 14 polylactic “arms” were obtained from tosylated CD via secondary hydroxyl groups by ROP with lactide (Figure 1A, R^2^; Adeli et al., 2008). Further ROP with 2-ethyl-2-oxazoline resulted in formation of block copolymers consisting of the CD core, PLA and seven polyoxazoline fragments. They formed micelles in chloroform and encapsulated the Congo red dye. The rate of the dye release increased with the length of both types of fragments (Adeli et al., 2008).

The CD substituted by a single amino group was functionalized with the PLA in the presence of *N,N*′-dicyclohexylcarbodiimide (DCC) as an activating agent (**1**, Figure 2B; Gao et al., 2005). The TG/DSC showed that grafting the PLA onto the CD reduced the glass transition temperature (T_g_). Introduction of a hydrophilic CD fragment led to significant increase in the biodegradation rate of the copolymer compared with unmodified PLA due to increased water diffusion into the copolymer. Resulting copolymers formed in aqueous solution negatively charged monodisperse particles of submicron size. The higher the content of CD in the copolymer the smaller their size and the lower their charge were (Gao et al., 2005). Micelles described could encapsulate BSA. Again, the higher the content of the CD fragment the more pronounced this ability was.

Block-copolymers **2** containing poly(ethylene glycol) fragments together with PLA were synthesized in a similar manner (Figure 2; Qiu et al., 2010). They could bind doxorubicin, an anticancer drug. A series of poly(ethylene glycol) copolymers with PLA was obtained by ROP of lactide with monomethoxypoly(ethylene glycol) as an initiator in the presence of stannous 2-ethylhexanoate (Kricheldorf and Serra, 1985). Terminal hydroxyl group of the polylactic unit was replaced by a carboxyl group by interaction with succinic anhydride in dioxane. Then, CD derivative containing seven ethylenediamine fragments was functionalized at primary amino groups by resulting acid in the presence of DCC and 4-(dimethylamino)pyridine (DMAP). Hybrids obtained could form micelles. Increase in the content of hydrophobic polylactide fragments decreased their critical micelle concentration and size. Most complete doxorubicin release was observed at pH 5.0. Cytotoxicity of the systems was observed only after the doxorubicin implementation. It significantly depended on the length of the polylactic chain. Longer chain showed higher cytotoxicity measured with the MCF-7/ADR cells.

21 polylactide fragments (Figure 1A, R^1^, R^2^) were introduced in the CD platform by ROP of D,L-lactide in the presence of tin octoate as catalyst (Yao et al., 2016). The substitution was observed at both primary and secondary hydroxyl groups of CD. The resulting 21-arm star copolymer was further modified with 2-(dimethylamino)ethylmethacrylate and 2-ethyl-2-oxazoline to corresponding block-copolymer. 2-(Dimethylamino)ethyl methacrylate block containing tertiary amino group was chosen to *in situ* reduction of the Au^3+^ ions to zero-valent gold via coordination—reduction mechanism without additional reductants. Monodisperse and structurally stable spherical unimolecular micelles containing CD and PLA as an inner core, poly(2- (dimethylamino)ethyl methacrylate) block as the middle layer and poly[oligo(2-ethyl-2-oxazoline)methacrylate block as the outer shell have been obtained by dialysis. The size of the micelles depended on the degree of polymerization of the monomers in the blocks and ranged from 20.9 to 28.5 nm (Yao et al., 2016; Zhang et al., 2016). The resulting products showed low cytotoxicity indicating advantages of the products as a nanoplatform for anticancer drug delivery and as contrast agents in computed tomography. They could also accumulate doxorubicin (Lin et al., 2017) separated between middle layer and micellar core (PLA block). The release of doxorubicin was pH dependent (max at pH 5.0) due to protonation of nitrogen atoms and accelerated PLA degradation in acidic media (Qiu et al., 2010). Doxorubicin loaded micelles were tested for antitumor efficiency against HepG2 cells (Yao et al., 2016; Lin et al., 2017).

The block-copolymer was also able to load imiquimod (synthetic immune response modifier) and plasmid DNA (Lin et al., 2016). Imiquimod was released most rapidly in acidic conditions in agreement with the above mentioned mechanism (Qiu et al., 2010; Lin et al., 2017). Cationic poly(2-(dimethylamino)ethyl methacrylate) block played a key role in formation of the complex with plasmid DNA.

In 2016, the synthesis of dendrimer like star polymer by click chemistry was reported (Tungala et al., 2016; Figure 1A, R^1^, Figure 2A). The CD primary hydroxyl groups were replaced by azide groups. Then three types of polymers (poly(methyl methacrylate), poly(*N*-isopropylacrylamide), PLA) were synthesized. The core was functionalized with polymethylmethacrylate and then re-azidated. The polylactide fragment was obtained by ROP of D,L-lactide at room temperature in the presence of 1,8-diazabicyclo[5.4.0]undec-7-ene (DBU) as a catalyst. Further, this fragment was modified by poly(*N*-isopropylacrylamide) to amphiphilic block-copolymer containing terminal alkyne group involved in the click reaction with an azide-polymethylmethacrylate block.

Pseudo-block copolymer based on the CD terminated poly(*N*-acryloylmorpholine) and adamantine-terminated linear poly(D,L-lactide) was obtained 2 years later (Ramesh et al., 2018) by ROP in the presence of DBU as a catalyst. The reaction was based on host-guest interactions, in which the inclusion complex of adamantane and CD moiety was formed. The obtained copolymer formed micelles with the size of 103 nm that were able to incorporate doxorubicin into the core. Doxorubicin release from the micelles was faster in acidic medium (Qiu et al., 2010; Lin et al., 2016, 2017).

## Hybrids With Tetrapyrroles

Porphyrins represent a unique class of synthetic and natural tetrapyrrole heterocyclic organic molecules (Imran et al., 2018), in which four pyrrole rings are linked together by methine bridges to form a plane macrocyclic structure with conjugated π-electrons (Figure 1B). Aromatic properties combined with the presence of a cavity make for them possible to bind various substrates.

In 2011, a star-shaped four-arm copolymer based on *meso*-tetra-(*p*-hydroxymethylphenyl) porphyrin was obtained by ROP of lactide in the presence of 2-[(2-dimethylamino-ethylimino) methyl] phenol) as a catalyst (Shieh et al., 2011; Hsu et al., 2012; Figures 1B, 2A). The photosensitizing properties caused by porphyrin macrocycle and ability to accumulate doxorubicin resulted in cytotoxic effect of the product toward MCF-7 cells resistant to doxorubicin.

A year later, poly(lactide-co-glycolide) with chlorine containing polymer **3** was synthesized using Steglich esterification (Lee et al., 2012; Figure 2). Further combination with a poly(lactide-co-glycolide) block-copolymer- polyethylene glycol gave water soluble product with low immune response and photo-sensitizing properties. Nanoparticles of about 160 nm were obtained which encapsulated magnetite required for high contrast magnetic resonance tumor imaging *in vivo*.

In 2016, poly(lactide-co-glycolide) fragment was covalently linked to porphyrin blocks by click reaction (Boix-Garriga et al., 2016; Figure 2A). The resulting copolymers **4** and **5** (Figure 2B) formed in aqueous solutions negatively charged nanoparticles of 114–148 nm in size, in which porphyrin fragments were located at the outer layer. They showed high photosensitizing ability to generate singlet oxygen, especially in the case of a hydrophilic porphyrin derivative **5**.

Four-armed copolymer was obtained from tetra(hydroxyethyl) terminated porphyrin and L-lactide by ROP in the presence of DMAP (Figure 1B; Dai et al., 2011, 2014a,b,c, 2015). Terminal hydroxyl groups of the copolymer were then modified with benzylsulfanylthiocarbonylsufanylpropionic acid (Dai et al., 2015). The poly(*N*-isopropylacrylamide) block was polymerized with corresponding monomer in the presence of azobisisobutyronitrile as an initiator. Resulting hybrid formed micelles in aqueous media able to change their shape (through cylinders to vesicles) at temperature near that of a body (37.2°C). It was also proved to be effective in generation of the singlet oxygen and inhibited BEL-7402 cancer cells. Similar hybrid with poly(ethylene glycol) block instead of poly(*N*-isopropylacrylamide) (Dai et al., 2014a) formed micelles in aqueous media that encapsulated doxorubicin released in acidic media (Qiu et al., 2010; Lin et al., 2016, 2017). The ability to generate singlet oxygen was retained.

Glycopolymers based on porphyrin containing four-arm copolymer were in 1-methyl-2-pyrrolidinone solution at 70°C obtained (Dai et al., 2014b,c). The copolymers generate singlet oxygen and fluorescence with a high quantum yield. Low dark cytotoxicity of the block-copolymers toward the COS-7 cells was shown (Dai et al., 2014b).

Based on tetrakis(4-aminophenyl) porphyrin, a four-arm star-shaped block copolymer including PLA fragment was obtained (Wang et al., 2015). First, D-α-tocopheryl polyethylene glycol 1,000 succinate was modified by D,L-lactide using the ROP in the presence of tin octoate. Further modification of block-copolymer with porphyrin was carried out in the presence of DCC and DMAP. For this purpose, terminal hydroxyl group of the polylactide block was first converted into carboxyl group with *N*-hydroxysuccinimide. Negatively charged nanoparticles up to 130 nm in size were obtained by nanoprecipitation from the copolymer to encapsulate cytostatic drug Docetaxel (Dai et al., 2014a) released back in acidic media (pH 5.0).

Nanocomposite based on carbon nanotubes and a four-arm PLA copolymer with zinc *p*-tetraaminophenylporphyrin was obtained by sonication due to non-covalent interactions caused by strong π-π interactions between carbon nanotubes and the porphyrin block of the copolymer (Li et al., 2016). Polymer with fully retained structure part was positioned outside the nanotubes.

## Hybrids With Calixarenes

Calixarenes are fully synthetic macrocycles of cup-shaped form produced by cyclic oligomerization of phenol with formaldehyde (Figure 1C; Gutsche, 1998). The presence of a hydrophobic cavity and the possibility of combining with hydrophilic substituents open up wide possibilities for their use as catalytic systems and receptors for recognition of numerous substrates (Gutsche, 1998; Ludwig and Dzung, 2002). Derivatives of classical calixarene and resorcinarene were first examples of hybrids with cyclophanes (Dria et al., 2012) obtained by ROP of lactide using stannous (II) octoate (Figure 1C). Using macrocycles with unsubstituted phenolic groups as “knot” elements, functionalization with lactide fragments proceeded slowly and not fully. Separation of the reaction centers from the macrocyclic platform showed possibility of the synthesis of target copolyesters with good yields and formation of completely substituted products (with 4 or 8 “arms”). The authors noted the effect of the macrocyclic center on thermal properties of the copolyesters. With smaller number of arms, both average molecular weight of the “arm” fragment and crystallinity degree of the sample decreased.

Thiacalixarene platform is favorably different from classical calixarene by the possibility of easy synthesis of different spatial isomers with intended position of the binding groups against cyclophane platform (Morohashi et al., 2006). Rather rigid fixation of the binding sites in the space allows high binding selectivity for different types of guests. All of this in combination with the non-toxicity of the macrocycle (Perret and Coleman, 2011) offers wide opportunities for its application. In 2018, amino derivative of *p*-*tert*-butylthiacalix[4]arene was firstly modified by L-lactide (Mostovaya et al., 2018) with preservation of the lactide fragment configuration. The resulting compound could bind dopamine. Fragments of the substituent but not the macrocycle itself played key role in the recognition. Endohedral complex was formed with dopamine coordinated outside the macrocycle cavity.

Modification of hydrazide derivatives of *p*-*tert*-butylthiacalix[4]arene in different conformations by LA led to formation of various products depending on the spatial loading of the reaction centers. In the case of their proximity (*cone* conformation), product **6** (Figure 2B) with four LA residues in oligolactide (OLA) fragments was obtained. More freely spaced substituents (*1,3-alternate*) were acylated with six residues (**7**, Figure 2B; Gorbachuk et al., 2018). The OLA obtained were able to self-association in methylene chloride. The associate size essentially depended on the spatial structure of thiacalix[4]arene stereoisomers. In *cone* conformation, all the OLA fragments were on one side of the macrocyclic rim. For the *1,3-alternate*, the OLA fragments interfere with the efficient packing of cyclophane, which results in much larger size of self-associates. The addition of silver nitrate to the copolymers resulted in disaggregation of self-associates.

Modification of the thiacalixarene platform by L-lactide could be carried out rather easily. However, a number of difficulties appeared in functionalization of the macrocycle directly with L-LA even for the most spatially unloaded *1,3-alternate* (Vavilova et al., 2019). “Knot” element of the tetracarboxyl macrocycle catalyzes the LA condensation with the formation of its pentamer. The replacement of carboxyl groups by ethoxycarbonyl did not lead to the positive result either. However, in the presence of MgSO_4_/*p*-toluenesulfonic acid mixture, the tetraester was modified by trilactide fragments over all ethoxycarbonyl groups (Figure 2A).

Introduction of a macrocyclic fragment significantly increased the decomposition temperature against that of unmodified pentalactide. Besides, resulting product was able to self-associate in polar solvents (Vavilova et al., 2019).

*p-tert*-Butylthiacalix[4]arene in *cone, partial cone*, and *1,3-alternate* conformations containing five lactide units in substituents was obtained by co-polycondensation in the melt (180°C) with pentameric LA (Gorbachuk et al., 2017; Gorbatchuk et al., 2017). Although a mixture of appropriate products was obtained, it was thermally more stable than unmodified penta-LA (Vavilova et al., 2019). In CH_2_Cl_2_, the mixture formed submicron particles by self-association. Their size significantly depended on the conformation of the “knot.” The largest associates were observed for *1,3-alternate* and the smallest ones for *cone*. Inverse relationship was found in water solution (Gorbachuk et al., 2017). Probably, decrease in the associate size with a higher solvent polarity could be explained by different packaging of the self-associates formed. They swell in dichloromethane by forming loose package, but do not swell in water. This significantly reduced particle size due to the denser packing of the copolyester molecules in micelles.

Largest oligolactide fragments were obtained by copolycondensation with penta-LA and tetra(penta-LA) derivatives of *p*-*tert*-butylthiacalix[4]arene (Gorbachuk et al., 2017) at 180°C in the presence of tin octoate (Mostovaya et al., 2019) (Figure 2A). In these conditions, lengthening of the chain of lactide residues to eight fragments in average occurred. However, the products obtained showed lower thermal stability (Gorbachuk et al., 2017; Vavilova et al., 2019). Probably, higher number of monomer units increased energy of inter- and intramolecular bonds. This explained higher decomposition temperature of unmodified octa-LA compared to penta-LA. The macrocyclic block acts as additional “loosening” element that weakens the bonds between OLA chains (Mostovaya et al., 2019). The resulting copolyesters form stable negatively charged submicron particles able to bind proteins. The coagulation was observed in the presence of positively charged lysozyme. In the case of BSA and hemoglobin, the associates retain submicron size exceeding 400 nm for *1,3-alternate*, and <200 nm for other isomers of the core. OLA modified by *partial cone* macrocycle interacted most efficiently with all the model proteins, while unmodified octalactide did not interact with them at all. Probably, interactions with biopolymers is mostly dependent on hydrophobic force between macrocyclic fragments of copolyesters and protein binding sites.

Self-association of the OLA modified thiacalixarenes has been used for assembling of electrochemical sensors where modifiers provided both accumulation of auxiliary agents and analytes. This resulted in significant improvement of analytical performance of the sensors. The permeability of the surface layer formed by drop-casting of the thiacalixarenes bearing five OLA fragments in each substituent was explored using electrochemical impedance spectroscopy and direct current voltammetry (Gorbatchuk et al., 2017). In both cases, negatively charged ferricyanide ion was utilized as redox probe. Its signals, i.e., cathodic current or charge transfer resistance, were sensitive to the charge density of the surface layer caused by carboxylic terminal groups of OLA fragments. The surface concentration and configuration of thiacalixarene core both influenced above parameters. Treatment of the OLA-thiacalixarene hybrids with AgNO_3_ followed by cathodic reduction of accumulated Ag^+^ ions resulted in formation of nanodendrites exerting electrocatalytic signals toward hydrogen peroxide, thiocholine, hydroquinone, tryptophan (Porifreva et al., 2018). As a result, their working concentrations have been decreased by more than one order of magnitude against bare glassy carbon electrode. OLA-thiacalixarene hybrids were also used as transducers in acetylcholinesterase sensors for determination of organophosphate pesticides and anti-dementia drugs exerting inhibitory effect on immobilized enzyme (Gorbatchuk et al., 2017; Shamagsumova et al., 2019).

## Conclusions

PLA has been modified by various macrocyclic fragments to obtain derivatives with the properties promising for drug delivery systems, photosensitizers in photodynamic therapy, and protein binding. These properties are determined by both the types of macrocyclic block and the number of lactide fragments in them. Variation in the above parameters as well as introduction of other substituents with functional groups can offer new opportunities for directional design of synthetic receptors and drug delivery systems with specific properties sensitive to the analyte properties and structural factors of macrocyclic core. Some of the advantages described have been already shown on the example of electrochemical sensors and biosensors with extended characteristics of drug, metabolite, antioxidant determination.

## Author Contributions

All authors listed have made a substantial, direct and intellectual contribution to the work, and approved it for publication.

### Conflict of Interest Statement

The authors declare that the research was conducted in the absence of any commercial or financial relationships that could be construed as a potential conflict of interest.
